# The effect of basketball intervention on executive function in children with autism spectrum disorders

**DOI:** 10.3389/fpsyt.2025.1720218

**Published:** 2025-12-29

**Authors:** Qi-Fan Wu, Wei-Min Cai, Jia-Qi Du, Feng Chang

**Affiliations:** 1 College of Physical Education, Anhui Normal University, Wuhu, China; 2 College of Sports Science and Health, Harbin Institute of Physical Education, Harbin, China

**Keywords:** basketball, children with autism, executive function, intervention training, non-invasive treatment

## Abstract

**Purpose:**

To investigate the effects of a basketball intervention on the development of executive functions in children with autism spectrum disorder.

**Methods:**

22 autistic children aged 6–12 years old in the elementary school section of the Wuhu Autism Association were randomly assigned into an experimental group (n=11) and a control group (n=11). The experimental group underwent basketball intervention training three times a week for 60 min each time for a total of 12 weeks, while the control group did not participate in any physical education course training, and only carried out usual daily routines. Inhibitory function, working memory and cognitive flexibility of the autistic children were tested before and after the trial.

**Results:**

After the basketball intervention, the experimental group showed significantly higher scores on the Stroop Color–Word Test, n-back task, and task-switching task were significantly higher than those of the control group (*P* < 0.01), which demonstrated the enhanced level of the experimental group in the three aspects of inhibitory control, working memory, and cognitive flexibility.

**Conclusion:**

Basketball training may potentially enhance executive function in children with ASD. Further discussion and mechanism analysis are warranted in future studies.

## 1 Introduction

Autism spectrum disorder (ASD) is a complex neurodevelopmental condition characterized by deficits in social communication, restricted interests, and repetitive behaviors. (1). Autism is a neurodevelopmental disorder. ASD has become one of the most rapidly increasing neurodevelopmental conditions worldwide, and the means of diagnosis and rehabilitation of autistic children has become a public health issue that requires urgent attention (2). The diagnostic tools and rehabilitation pathways for autistic children have become an urgent public health concern. Executive functioning is a set of cognitive processes that allow individuals to consciously and effectively control their thoughts and behaviors, and covers three core subfunctions: inhibitory control, working memory, and cognitive flexibility (3). Executive functioning is a set of cognitive processes in which individuals consciously and effectively control their thinking and behavior. Numerous studies have demonstrated that executive functioning is an important predictor of physical and mental health, academic performance, social interaction, and quality of life (3–35). The following is an example of an intervention in executive functioning during childhood. Intervening in executive functions during childhood can lay a solid foundation for future learning and life. Children with ASD often present with a higher rate of comorbidities compared with typically developing peers. (4). Research on cognitive executive functioning has found that executive dysfunction has become a common symptom in more than 85% of children with autism (5). The neural mechanisms underlying ASD involve altered functional connectivity within the default mode network (DMN) (6). Impairment of executive functioning in children with ASD not only leads to difficulties in motor skill formation and delays in coordination and reaction time, but can also have a significant impact on their physical and mental health (7). The impact on their physical and mental health can be substantial.

According to Piaget’s cognitive development theory, motor activity and cognitive development are interrelated, with sensorimotor experiences serving as a foundation for higher cognitive processes. (8). The components of executive functioning are responsible for planning and efficiently executing task goals, and when an individual is accomplishing a complex task, executive functioning coordinates various cognitive processes to ensure that the cognitive system accomplishes the general control of the task goal (9). Executive functioning in children with ASD is an important indicator of healthy childhood development (10). Executive functioning is an important indicator of healthy childhood development in children with ASD.

Prior research has indicated that basketball had a significant effect on both executive functioning and socialization in young children (11). The results of this study show that basketball has a significant effect on both executive functioning and social skills. The level of executive functioning is generally negatively correlated with the core symptoms of autism, i.e., the higher the executive functioning, the lower their core symptoms correspondingly. The feasibility of basketball as an intervention in enhancing executive function and reducing core symptoms in ASD patients is demonstrated. Current intervention approaches for children with ASD include pharmacological, behavioral, music, play, and sports-based therapies, both domestically and internationally. Basketball is a dynamic, skill-oriented sport characterized by open movement patterns, competitiveness, and enjoyment. In basketball, participants are required to make adjustments in response to changes or stimuli in the external environment while accomplishing the set goals. Consequently, basketball requires the integration of perceptual, cognitive, and motor systems, stimulating neural activation and enhancing cognitive engagement (12). Currently, most domestic and international studies on basketball interventions are applied to enhance the social and physical abilities of children with autism, while fewer focus on basketball as an intervention to enhance the executive function of children with autism.

In this study, autistic children were recruited as the experimental subjects to explore the impact of basketball sports intervention on their executive function behavioral performance. The aim was to contribute research insights for developing rehabilitation strategies targeted at executive dysfunction within the autistic population. Thus, the findings of this investigation could serve as valuable references for advancing rehabilitation means specific to executive dysfunction in autistic children.

## 2 Research objects and methods

### 2.1 Objects of study

**Figure 1:**
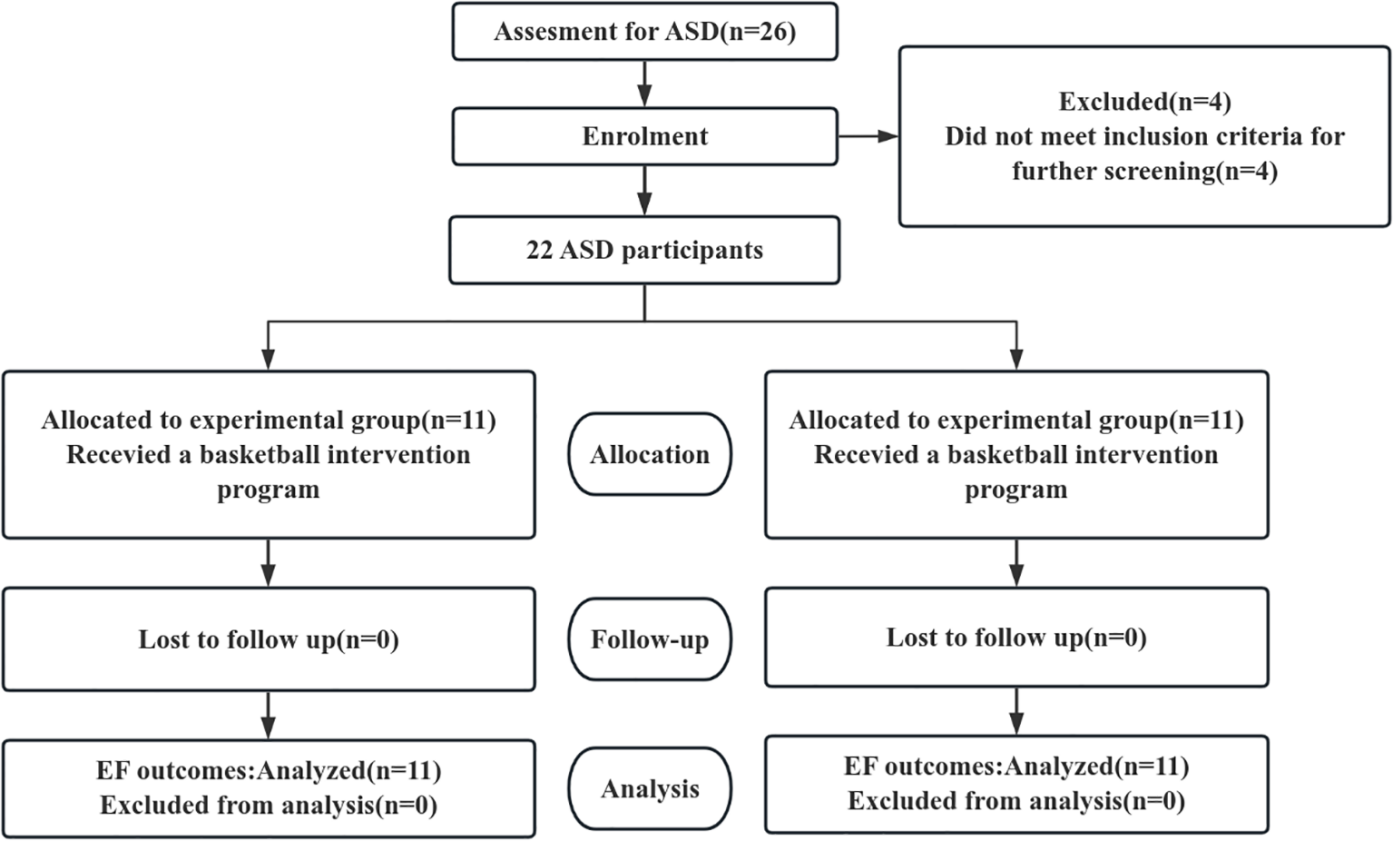
CONSORT diagram of the experimental protocol.

Despite prior sample size estimates (G*Power 3.1, effect size Cohen’s f = 0.35, α = 0.05, power = 0.80), detecting a moderately large interaction effect would require at least 18–22 participants per group. However, recruiting children with autism proved extremely challenging due to strict inclusion criteria, parental consent constraints, and geographical limitations. To compensate for potential statistical power limitations, this study reported observed power, Hedges’ g effect size, and 95% confidence intervals, and conducted multiple sensitivity analyses to validate the robustness of the results. Participants for this study were recruited by the author at the local autism association. Inclusion criteria: age 6–12 years, possession of a diagnostic certificate of autism from the association or a tertiary hospital, and no basketball training. Exclusion criteria: physical movement disorders, epilepsy or other sudden illnesses, violent tendencies, audiovisual disorders. Exclusion and exclusion criteria: inability to participate in the full training program, voluntary request to withdraw from the study in the middle of the study. The above criteria are intended to exclude the influence of various unforeseen factors on the experimental intervention. According to the above criteria, a total of 26 children with autism were recruited, of which 4 parents indicated that the intervention site was far away and could not attend on time, so the final number of autistic children participating in the study was 22. Using computer-generated random sequences, subjects were assigned to the experimental and control groups in a 1:1 ratio via block randomization. The random sequence was generated by an independent statistician not involved in subject recruitment or allocation. Allocation concealment were sealed in sequentially numbered, opaque envelopes and executed within the centralized randomization system, with 11 in the control group (7 males/4 females) and 11 in the experimental group (8 males/3 females). The experimental group participated in basketball training for 12 weeks, 3 times per week, 60min/time,. Meanwhile the control group did not participate in any sports activities of the same type. Written informed consent was obtained from the parents or legal guardians of the study participants prior to their participation in the study. All methods were performed in accordance with the relevant guidelines and regulations. For research involving human participants, the study was conducted in accordance with the Declaration of Helsinki. The demographics of the experimental subjects were analyzed in **Table 1**. The overall experimental design process is shown in **Figure 1** below.

**Table 1:** Demographic information of experimental subjects.

Clusters	Age (year)	Height (m)	Weight (kg)	BMI
Experimental group (n=11)	8.25 ± 1.04	1.32 ± 0.05	34.25 ± 2.12	19.56 ± 1.03
Control group (n=11)	8.75 ± 1.98	1.29 ± 0.05	35.38 ± 4.14	21.56 ± 3.51

### 2.2 Research methodology

#### 2.2.1 Design and quality control of intervention programs

This study was based on the multi-path theory of sports to promote children’s brain intelligence development (34) and also refers to the existing research results, combines the characteristics and development trend of autistic children (13). A structured basketball training program was designed. The course plan is shown in **Table 2**. Each basketball session consisted of three parts: “warm-up, core basketball practice, and cool-down activities.”, and the intensity of the basketball training was kept at a medium load. The single-session training plan is shown in **Table 3**. Three subjects were randomly assigned to wear a heart-rate monitoring bracelet (Huayin Medical YX-023YWHSX) with a heart rate test function in each lesson, and their heart rate was kept at 70% HR_max_(208 - 0.7 × Age), valid in ASD as it relies on age, not affected by neurodevelopmental differences (14). All training sessions were conducted as scheduled. Heart rate ranges displayed on heart rate monitors were checked after each session to ensure heart rates remained within preset levels. Attendance was 100% with no adverse events reported. The coach-to-participant ratio during training sessions was 1:2.75 (4 coaches: 11 participants). All coaches held teaching certificates certified by the Education Bureau. All volunteers received one week of standardized training from the Autism Association before study commencement.

**Table 2:** Basketball intervention class program.

Annular ring	Training content	Timing
Preliminary part	Running with a ball, static/dynamic stretching, sports trivia games	10min
Fundamental part	Basketball skill acquisition, physical fitness exercises	40min
1–3 weeks	Basketball skills: dribbling with left and right hands in place, switching hands in place, one-handed v-dribbling Physical fitness: rope ladder footwork exercises, agility ball exercises	
4–6 weeks	Basketball skills: review, traveling dribble (straight/curve), shooting from the floor under the basket	
	Physical: elbow and knee braces, full-court slide, forward and backward crossovers	
7–10 weeks	Basketball skills: review, change of direction in front of the body between lines, two-handed chest pass and catch	
	Physical fitness: continuous one-legged jump, high leg run, static squat against a wall	
11–12 weeks	Basketball skills: review, one-handed shoulder shot, 1v1, 3v3 games	
	Physical fitness: Plank support, crunch run, arrow squat	
End part	Finishing activities: relaxing muscles, stretching exercises	10min

ASD Children were dynamically divided into 3–4 ability groups each session, and coaches (ratio 1:2–1:3) adjusted task difficulty (ball size, distance, rules complexity, and assistance level) in real time according to each child’s performance and emotional state on that day.

**Table 3:** Baseline characteristic balance test.

Variable	Experimental group	Control group	SMD
Age (years)	8.25 ± 1.04	8.75 ± 1.98	−0.316
Height (m)	1.32 ± 0.05	1.29 ± 0.05	0.758
Weight (kg)	34.25 ± 2.12	35.38 ± 4.14	−0.342
BMI	19.56 ± 1.03	21.56 ± 3.51	−0.772
CARS	35.41 ± 1.44	35.58 ± 0.41	−0.147
Sex (Male)	8 (72.7%)	7 (63.6%)	−0.254
Stroop accuracy (consistent)	90.00 ± 2.89	89.45 ± 3.42	0.174
Stroop accuracy (inconsistency)	80.81 ± 2.68	77.80 ± 2.41	1.183
Stroop reaction time (consistent)	1514.29 ± 144.03	1525.29 ± 111.53	-0.085
Stroop reaction time (inconsistent)	1817.42 ± 194.71	1891.06 ± 189.70	-0.383
1-back accuracy	77.29 ± 4.72	76.69 ± 3.89	0.139
1back reaction time	1579.33 ± 83.45	1599.54 ± 64.49	-0.271
2-back accuracy	64.83 ± 2.41	63.67 ± 4.35	0.327
2-back reaction time	2511.21 ± 218.69	2662.18 ± 145.90	-0.812
Task-switching accuracy	83.29 ± 2.01	81.97 ± 1.41	0.756
Task-switching reaction time	1797.64 ± 75.13	1791.53 ± 88.37	0.075

#### 2.2.2 Selection of inhibitory control indicators

**Figure 2:**
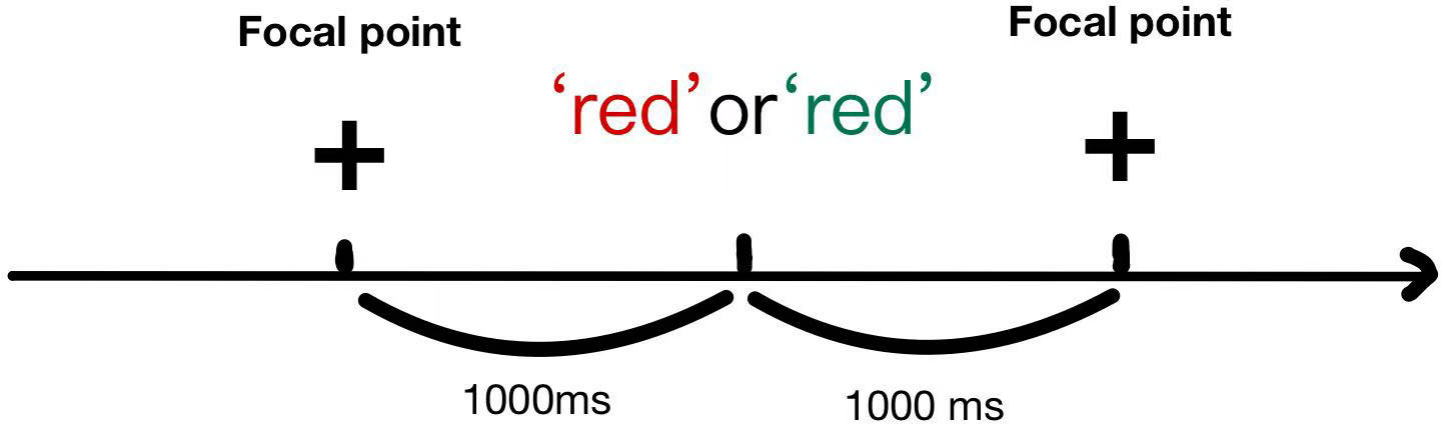
Schematic diagram of the stroop task.

Inhibitory control reflects the subject’s ability to inhibit irrelevant stimuli, and the inhibitory control test is based on the stroop color-word task. At the beginning of the test, a black focal point “**+**” appeared on the screen, and then two Chinese characters “red” or “green” were randomly presented, and the color of the Chinese characters could be red or green. The Stroop test procedure is shown in **Figure 2**. And the Chinese character version of the Stroop test has been demonstrated to be feasible in testing children aged 6–12 in previous studies (15, 16). When the meaning of the Chinese character is the same as the color of the Chinese character, it is a consistent condition, otherwise it is an inconsistent condition. Each Chinese character was followed by a keystroke response, with a stimulus interval of 1,000–1500 ms. The task consisted of 32 trials, and the metrics were reaction time, which included the reaction time of the incongruent condition, the reaction time of the congruent condition, and the difference between the reaction times of the two conditions; and accuracy, which included the accuracy rate of the incongruent condition and the accuracy rate of the congruent condition (17). 

#### 2.2.3 Selection of working memory indicators

The working memory of subjects was assessed using an n-back task, as described by Davidson, (18, 18). Specifically, two loading conditions were employed: n=1 and n=2. In the 1-back condition, participants were instructed to determine if the currently presented letter matched the immediately preceding one. Conversely, in the 2-back condition, they were required to ascertain if the current letter matched the one presented two positions back. Prior to the formal testing phase, which comprised 16 trials. Between each trial, a blank screen was displayed for a duration ranging from 500 to 1000 milliseconds. Participants responded by pressing a key, and their response times and accuracy rates were recorded for both conditions.

#### 2.2.4 Selection of cognitive flexibility indicators

A task-switching task was used to test subjects’ level of cognitive flexibility, where a sun or moon pattern was presented on a screen and switched according to different rules (19). When the sun pattern appeared, the subject was asked to press a button in the same direction. When the sun pattern appeared, subjects were asked to press a button in the same direction; when the moon pattern appeared, subjects pressed a button in the opposite direction, and each pattern appeared 10 times randomly. Before each presentation of the pattern in the formal test, the screen would show a 500-ms gaze point, followed by a 3000-ms stimulus, and then a 1,000-ms blanking period, and the above process was repeated 10 times. The average reaction time and accuracy were recorded (20).

#### 2.2.5 Selection of behavioral indicators

Subjects were retested using the CARS scale, the Autism Rating Scale (CARS), developed by Schopler (21) et al. was developed for individuals with ASD from childhood through adulthood. The scale consists of 15 assessment items, each of which is rated on a scale ranging from 1 to 4, with a total score of 60. The score results reflect the severity of autism: scores below 30 indicate no autism symptoms, scores between 30 and 35 are considered mild to moderate autism, and scores above 35 indicate severe autism. The present study was a homogeneous test of the degree of illness in the 2 groups of children based on their CARS scores.

#### 2.2.6 Experimental procedures and variable control

This experiment employed a single-blinded control method to minimize bias, meaning that neither the basketball intervention coaches nor the participants were aware of the group assignments or the purpose of the experiment. The stimulus material was presented using the built-in E-prime 3.0 software on a Lenovo laptop (15-inch, 1920×1080 resolution). Data collection is performed automatically by machines. All testing sessions were conducted in a quiet, well-lit, and standardized psychological assessment environment. Only the researcher and an assessor holding professional assessment qualifications were present during evaluations to prevent interference from other irrelevant stimuli. Before the formal test begins, The tester explains the experimental procedure in a gentle and concise tone, repeating it 2–3 times to ensure the subject understands. And meanwhile, each participant will complete eight pre-tests. Participants achieving less than 50% accuracy during pretests were excluded from formal testing.

### 2.3 Statistical methods

All statistical analyses in this study were performed using the R software (version 4.4.2). The primary packages employed included lme4, lmerTest, emmeans, and effectsize. First, to assess randomization balance, standardized mean differences (SMDs) were calculated for all baseline demographic variables and cognitive task measures. Intergroup comparability was determined using |SMD| < 0.8 as the criterion. To evaluate intervention effectiveness, linear mixed-effects models served as the core analytical approach. For each cognitive task measure, the following model was constructed:

lmer(Score ~ Time * Group + Age + Sex + bmi +CARS+ (1 | ID))

Fixed effects included: Time × Group interaction term: Directly assessed intervention effect, i.e., whether the change trend from pre-test to post-test in the experimental group differed significantly from the control group. Covariates: The model simultaneously adjusted for age, sex, CARS and BMI to control for their potential influence on cognitive outcomes. Random effects comprised participant-specific intercepts (1 | ID) to account for repeated measures characteristics within the same participant. Model parameter significance was tested using Satterthwaite’s approximate degrees of freedom method provided by the lmerTest package. For indicators showing significant interactions, Hedges’ g effect sizes and their 95% confidence intervals were further calculated. This effect size was standardized based on the residual standard deviation from the ANCOVA model adjusted for covariates and underwent small-sample correction to provide a more accurate effect size estimate.

To assess the robustness of the findings, we conducted a systematic *post-hoc* sensitivity analysis: a simplified mixed model without adjusting for any covariates; a covariance analysis model adjusting only for baseline scores; and a re-run of the primary mixed model after excluding extreme outliers using the interquartile range method. Additionally, this study assessed the risk of Type II errors for indicators with insufficient statistical power and pre-specified a single primary outcome measure based on theoretical importance and statistical evidence to guard against multiple comparison issues.

## 3 Results

### 3.1 Difference test of each index between experimental group and control group before intervention

Baseline characteristic analysis revealed no significant differences in the distribution of any indicators between the two groups (all |SMD| < 0.8), indicating good comparability between groups. Furthermore, no significant intergroup differences were observed in all cognitive task metrics at baseline, providing a solid foundation for subsequent analysis of intervention effects. Despite random assignment, a small sample size (n=11 per group) resulted in some baseline imbalance in 2-back reaction time (SMD = -0.812). This reflects the random variability commonly encountered in small-sample studies. To control for the potential impact of this baseline imbalance on outcome estimates, we adjusted for key covariates affecting cognitive performance—age, gender, and BMI—in all primary analyses (linear mixed models). Notably, the baseline 2-back reaction time itself was part of the outcome variable. Its ‘change’ was directly modeled and analyzed through the ‘time × group interaction’ in the mixed model. This approach outperformed an ANCOVA model treating baseline as a covariate, more effectively addressing such predictor imbalance issues. 

### 3.1 Mixed-effects model results of inhibition before and after intervention

As shown in **Table 4**, the intervention group exhibited significant group × time interaction effects across all Stroop task metrics (all *P* < 0.001). Accuracy improvement was particularly pronounced under the incongruent condition (Adjusted mean difference = 6.71, Hedges’ g = 3.02), accompanied by a significant reduction in reaction time (Adjusted mean difference = −165.80, Hedges’ g = −1.94). Results under the congruent condition also supported significant improvements in executive control and processing speed for the intervention group. These findings indicate that the intervention effectively enhanced participants’ inhibitory control and conflict resolution abilities.

**Table 4:** Mixed-effects model results for stroop task measures.

Inhibitory control indicator	Mixed model results (time × group interaction)				Effect size		Descriptive statistics	
	Interaction item	*P*-value	Adjusted mean difference	95% CI	Hedges’ g	Hedges’ g 95% CI	Pre-test (mean ± SD)	Post-test (mean ± SD)
Stroop accuracy (Inconsistent) (%)	Timepost: Group	< 0.001	6.71	[3.46, 9.96]	3.02	[1.59, 4.45]	EG:80.81 ± 2.68 CG:77.80 ± 2.41	EG:87.59 ± 2.85 CG:77.50 ± 2.95
Stroop reaction time (Inconsistent) (ms)	Timepost: Group	< 0.01	-165.80	[-283.31, -48.29]	-1.94	[-3.13, -0.75]	EG:1817.43 ± 194.71 CG:1891.06 ± 189.70	EG:1571.66 ± 175.89 CG:1812.28 ± 99.15
Stroop accuracy (Consistent) (%)	Timepost: Group	< 0.001	4.54	[3.84, 5.24]	6.92	[4.33, 9.51]	EG:90.00 ± 2.89 CG:89.45 ± 3.42	EG:93.90 ± 2.35 CG:89.21 ± 2.00
Stroop reaction time (Consistent) (ms)	Timepost: Group	< 0.001	-319.27	[-4.18.23,-220.32]	-3.41	[-4.95, -1.88]	EG:1514.29 ± 144.03 CG:1525.29 ± 111.53	EG:1311.43 ± 149.28 CG:1627.24 ± 132.17

### 3.2 Mixed-effects model results of working memory before and after the intervention

As shown in **Table 5**, the intervention group exhibited significant group × time interaction effects across all Stroop task metrics (all *P* < 0.001). The improvement in accuracy under the incongruent condition was particularly pronounced (Adjusted mean difference = 6.71, Hedges’ g = 3.02), accompanied by a significant reduction in reaction time (Adjusted mean difference = −165.80, Hedges’ g = −1.94). Results under the congruent condition also supported significant improvements in executive control and processing speed for the intervention group. These findings indicate that the intervention effectively enhanced participants’ inhibitory control and conflict resolution abilities.

**Table 5:** Mixed-model results for working memory (n-back task) metrics.

Working memory indicator	Mixed model results (time × group interaction)				Effect size		Descriptive statistics	
	Interaction item	P-value	Adjusted mean difference	95% CI	Hedges’ g	Hedges’ g 95% CI	Pre-test (mean ± SD)	Post-test (mean ± SD)
1-back accuracy (%)	Timepost: Group	< 0.001	6.94	[3.26, 10.63]	2.18	[0.94, 3.41]	EG:77.29 ± 4.72 CG:76.69 ± 3.89	EG:82.04 ± 4.84 CG:74.73 ± 2.94
1-back reaction time (ms)	Timepost: Group	< 0.001	-198.39	[-254.97, -141.79]	-3.76	[-5.39, -2.13]	EG:1579.33 ± 83.45 CG:1599.54 ± 64.49	EG:1433.38 ± 60.54 CG:1635.91 ± 51.04
2-back accuracy (%)	Timepost: Group	< 0.01	5.52	[2.67, 8.37]	2.51	[1.20, 3.82]	EG:64.83 ± 2.41 CG:63.68 ± 4.35	EG:68.76 ± 2.28 CG:62.43 ± 3.02
2-back reaction time (ms)	Timepost: Group	< 0.001	-283.94	[-387.04, -180.83]	-3.12	[-4.59, -1.66]	EG:2511.21 ± 218.69 CG:2662.18 ± 145.90	EG:2268.53 ± 218.41 CG:2720.60 ± 90.78

### 3.3 Mixed-effects model results of cognitive flexibility before and after intervention

The results for task-switching ability are shown in **Table 6**. Regarding accuracy, the intervention group demonstrated a highly significant improvement effect (*P* < 0.001, Hedges’ g = 7.24), representing the largest effect size among all measures, indicating that the intervention significantly enhanced cognitive flexibility. However, while improvements in task-switching reaction times were statistically significant (*P* < 0.05), the effect size was small (Hedges’ g = -0.95). Moreover, its 95% confidence interval was broad, encompassing zero (-1.99 to 0.08), suggesting that the intervention’s effect on reaction times was unstable. 

**Table 6:** Mixed model results for task switching metrics.

Cognitive flexibility indicator	Mixed model results (time × group interaction)				Effect size		Descriptive statistics	
	Interaction item	*P*-value	Adjusted mean difference	95% CI	Hedges’ g	Hedges’ g 95% CI	Pre-test (mean ± SD)	Post-test (mean ± SD)
Task-switching accuracy (%)	Timepost: Group	< 0.001	5.70	[4.75, 6.64]	7.24	[4.55, 9.93]	EG:83.29 ± 2.01 CG: 81.98 ± 1.41	EG: 87.49 ± 1.40 CG: 80.91 ± 0.80
Task-switching Reaction time (ms)	Timepost: Group	< 0.05	-67.88	[-144.49, 8.74]	-0.95	[-1.99, 0.08]	EG:1797.64 ± 75.13 CG:1791.53 ± 88.37	EG:1730.45 ± 87.25 CG:1803.51 ± 100.22

### 3.4 Trends in indicators before and after intervention

**Figure 3:**
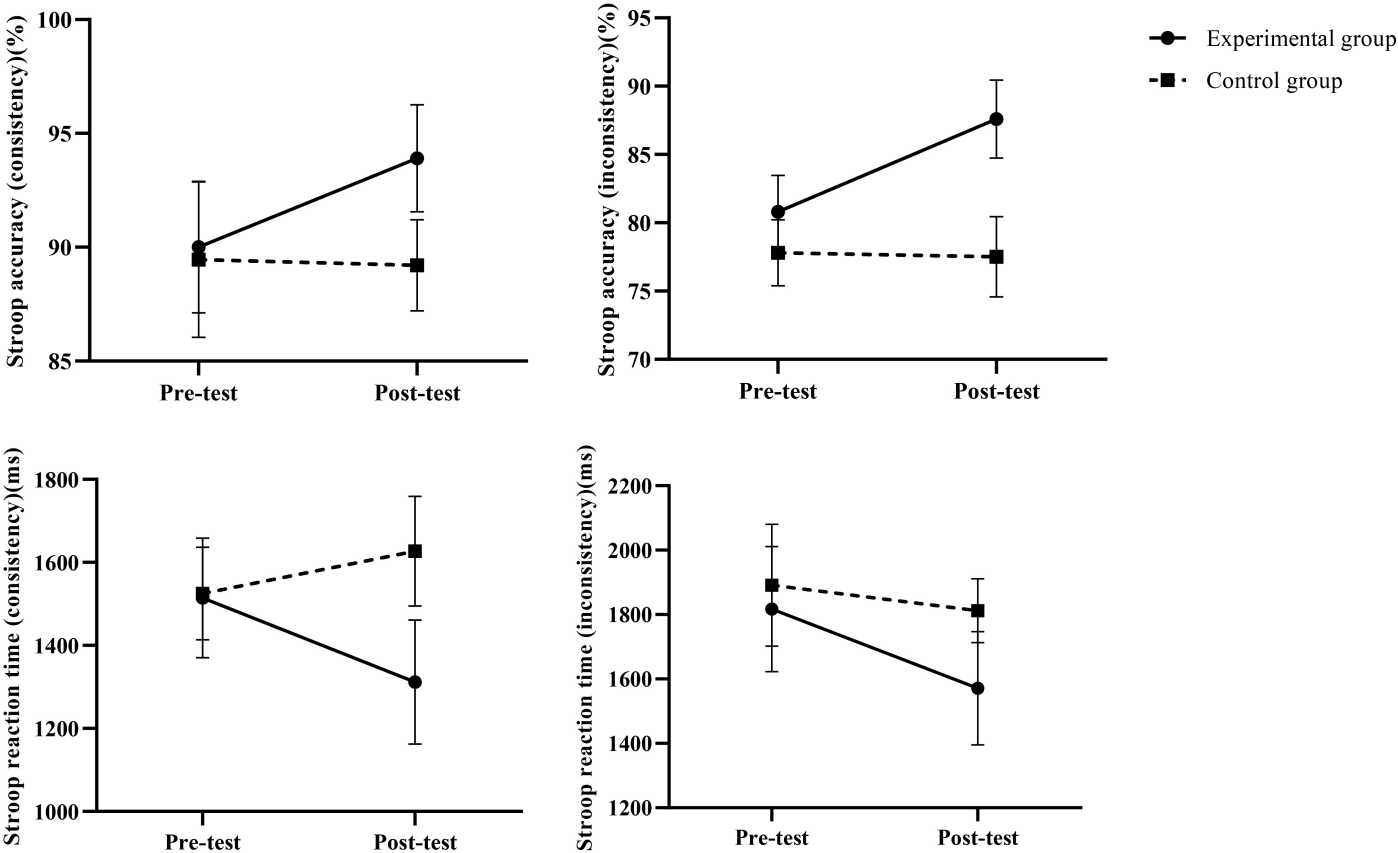
Changes in inhibitory capacity before and after intervention.

**Figure 4:**
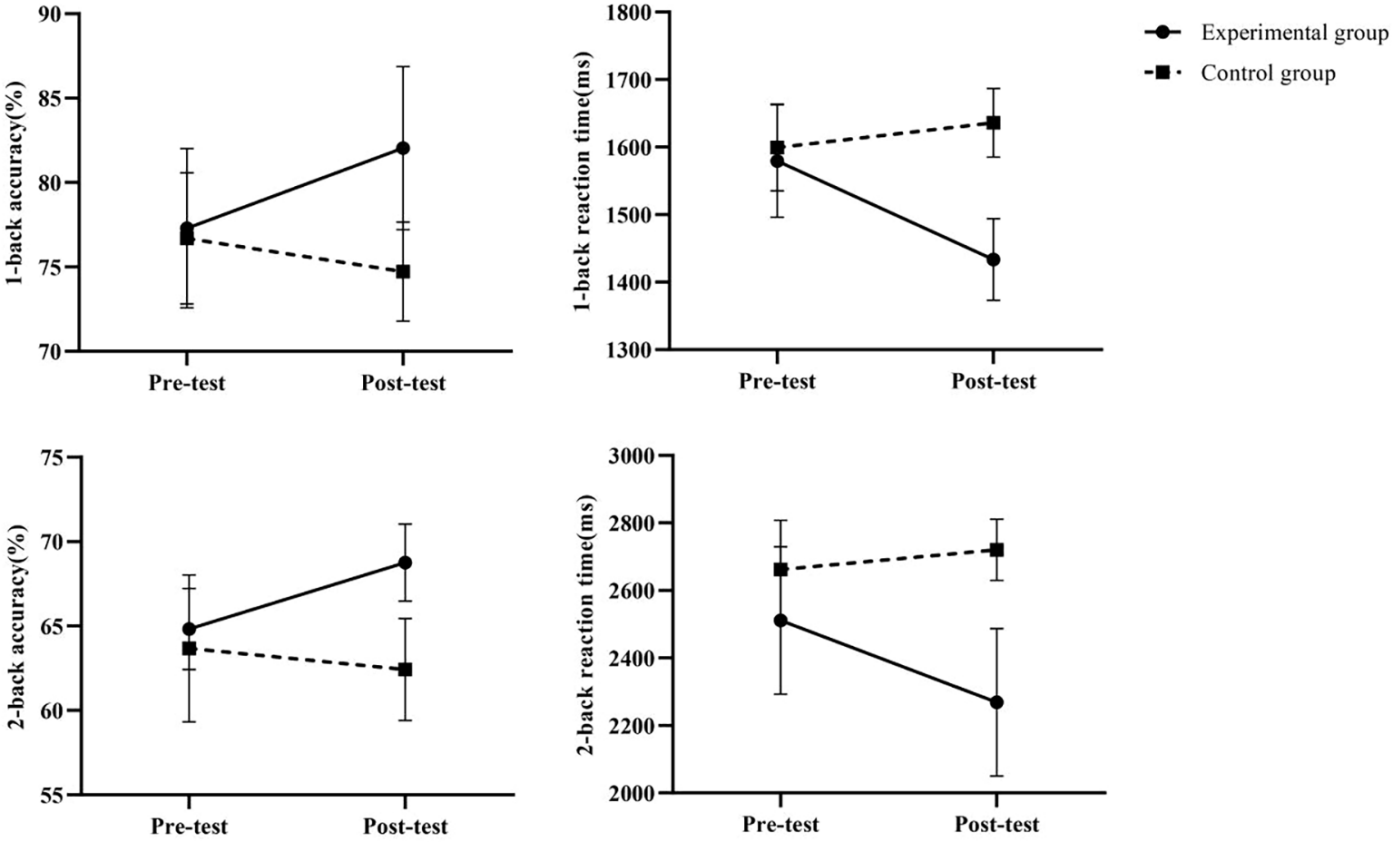
Changes in working memory before and after intervention.

**Figure 5:**
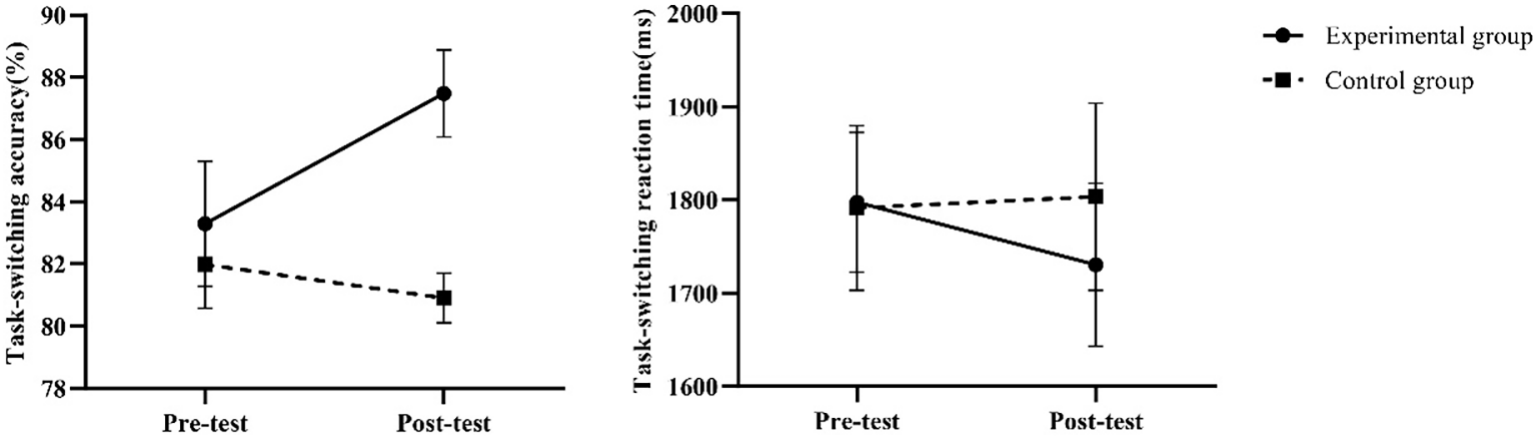
Changes in cognitive flexibility before and after intervention.

By assessing the robustness of our findings through sensitivity analyses. Cognitive task metrics maintained statistical significance across three distinct sensitivity analysis approaches (all *P* < 0.05), fully consistent with the primary analysis results. Specifically: Unadjusted model: Time × group interactions remained significant for all metrics (*P* < 0.05);ANCOVA model adjusting for baseline values: Group effects remained significant across all measures (*P* < 0.05);Outlier exclusion analysis: The pattern of results for all measures remained unchanged after excluding 1–5 outliers. Notably, even the task-switching reaction time—which exhibited a relatively small effect size (Hedges’ g = -0.95)—demonstrated stable statistical significance across sensitivity analyses. These findings indicate that the study’s conclusions possess high robustness, independent of specific analytical method selection. Visualizations of the indicators before and after the intervention are shown in **Figures 3**–**5**.

Type II error risk analysis indicates that the statistical power for detecting cognitive improvement varies across measures given the current sample size (n=11 per group). Except for task-switching reaction time, all measures exhibit statistical power exceeding 95%, suggesting high reliability for these results. Notably, the statistical power for task-switching reaction time is only 42.5%, suggesting a potentially high risk of Type II error for this measure—i.e., the possibility of failing to detect a genuine intervention effect due to insufficient sample size. However, despite its relatively low statistical power (42.5%), the consistency observed in sensitivity analyses provides supporting evidence for this marginally significant result.

## 4 Discussion

The results of this study showed that the experimental group showed significant improvement in performance on several cognitive task indicators after the intervention, forming a significant difference from the control group, especially in the reaction time and accuracy rate of the Stroop task, the reaction time of the 1-back and 2-back tasks, and the accuracy rate of the task-switching task; the effect of the intervention was particularly significant. Based on this, it can be seen that a 12-week basketball intervention training had a positive effect on the executive functioning of children with autism (7, 36) showed that a small basketball training program as an intervention had a positive effect on all three sub-dimensions of executive functioning in children with autism and mental retardation, confirming that sports interventions can have a positive effect on executive functioning. which also confirmed that exercise intervention can improve executive functioning in children with autism, consistent with the findings of this study. The lack of significant between-group effects in task-switching reaction times may stem from the basketball task’s relatively fewer rapid and unpredictable rule changes compared to the sun/moon task-switching paradigm used in the test. This limitation likely constrained its transfer effects on reaction time measurements (despite a significant improvement in accuracy).

This study not only statistically confirmed the significant enhancement of executive function in children with autism following a 12-week basketball intervention but, more importantly, demonstrated that the improvement reached a level of substantial practical significance. The experimental group exhibited increased accuracy and reduced reaction times across three core subcomponents: inhibitory control, working memory, and cognitive flexibility (15). These changes surpassed the commonly used “practical significance” threshold in cognitive intervention studies for children with autism. Particularly noteworthy is the largest improvement in cognitive flexibility accuracy. This indicates that children participating in basketball training saw their correct response rate when facing sudden rule or task changes rise from a level “close to random” to one approaching that of typically developing children. Such a degree of improvement is highly likely to transfer to scenarios like classroom listening, social turn-taking, and daily living skills, demonstrating strong ecological validity (22–33).

The basketball training program in this study was intended to enhance physical fitness and foster the development of skill proficiency patterns. Each intervention session emphasized the improvement of mobility, ball handling (including dribbling), passing and receiving, as well as shooting drills. Physical fitness gains were achieved through footwork agility drills and an array of static exercises within the physical training component. Furthermore, the two coaches offered consistent guidance utilizing both teaching aids and verbal directions during the intervention sessions. They integrated rewards, punishments, and games to sustain motivational engagement in the task objectives among children with ASD, thereby promoting enhanced attentional stability and further refining their motor memory abilities (21).

The four coaches provided continuous guidance through teaching aids or verbal instructions, combined with reward mechanisms and games, which helped children with ASD maintain motivational sensitivity to task goals and increase attentional stability while further enhancing motor memory skills. Numerous studies have found that long-term regular exercise can lead to structural changes in the brain, modulate neural network links, and improve the brain’s neural adaptive and protective abilities (38).

The footwork combined with the disguised movement The footwork combined with disguised movement training program requires the trainer to coordinate body posture and motor movements while accomplishing the goal, requiring a high degree of concentration, emphasizing body and ball control and rapid response, thus improving reaction time and promoting cognitive development in children with ASD (22). Some studies have shown that basketball training has a positive effect on the development of young children’s gross motor level (32) and that the level of gross motor development is highly positively correlated with executive functioning (23) In the present study, the experimental group was trained in basketball. In the present study, the experimental group showed a significant increase in executive functioning after 12 weeks of basketball training, which may be explained by the shared neurological basis of cognitive-motor development (24). Children with ASD promote cognitive level development by exploring and discovering the external environment through perceptual-motor crossover (31). The present study set up several compound movements during the intervention, such as dribbling a ball with one hand while pushing the handle cone with the other hand. Compound training places children with ASD in a dynamic and unpredictable environment, prompting them to respond in a timely manner, awakening the parasympathetic nerves in their brains of children with ASD, and producing brain-derived trophic factors that promote neurological plasticity (30). This environment may be important for improving executive functioning in children with ASD (25). Simultaneously, the curriculum was designed to include competitions and games, and the tense and exciting environment, as well as the desire to win, stimulated the brain to synthesize and release norepinephrine and dopamine (37), which increases the speed at which neurons process information and improves brain activity (26). It causes microstructural changes in the white matter of the brain, leading to a closer functional network between the peri-striatal brain regions and promoting the development of working memory (27). The brain is also a good starting point. In addition, from an anatomical perspective, the improvement of executive functioning in children with ASD is due to the modulation of organ biological responses by basketball intervention, which positively affects the brain and neurodevelopment (28). However, basketball interventions also have clear limitations: ① They require significant resources in terms of venues (indoor/outdoor basketball courts), equipment (basketballs, marker cones), and professional coaches; ② They necessitate at least 6–8 participants simultaneously to create competitive and cooperative dynamics, making organization more challenging than swimming, running, or yoga; ③ They may present initial barriers for some children with autism who have severe motor impairments or social anxiety (29).

Therefore, basketball is more suitable as a routine group activity in schools or rehabilitation centers for children with mild to moderate autism, basic motor skills, ages 6–14, and high parental cooperation. For children with severe autism or significant motor coordination deficits, individualized activities like swimming or cycling should be prioritized, or a 4–8 week foundational movement training program should precede basketball. Future exploration of a “tiered progression” exercise prescription model could maximize the complementary benefits of different sports formats.

## 5 Research limitations and prospects

The current study encountered challenges such as a limited intervention duration, modest sample size, and absence of sustained efficacy tracking post-intervention. Although statistically tested, the research findings may satisfy robustness criteria, but the small sample size may have overestimated the effect size. Future studies should replicate these findings in larger samples. The passive control (no PE) may confound effects via unequal attention/socialization, and an active control (e.g., non-basketball activity) would better isolate basketball’s specificity. Additionally, the involvement of four coaches in the intervention may result in overcrowding and occasional oversight of participants, potentially compromising the study’s generalizability and reliability. To address these issues, future research should consider prolonging the intervention period, augmenting the sample size, initiating continuous monitoring, and increasing the number of assistants during the post-intervention follow-up period. These enhancements would further refine the intervention experiment, ultimately offering more comprehensive insights into the treatment of autistic children.

## 6 Conclusion

Basketball training may potentially enhance executive function in children with ASD. However, due to the brief follow-up period, limitations in sample size leading to issues with effect separation, and constraints in blinding control, further discussion and mechanism analysis are warranted in future studies.

## Data Availability

The original contributions presented in the study are included in the article/supplementary material. Further inquiries can be directed to the corresponding author.

## References

[1] Christensen DL Baio J Van Naarden Braun K Bilder D Charles J Constantino JN . Prevalence and characteristics of autism spectrum disorder among children aged 8 years–autism and developmental disabilities monitoring network, 11 sites, United States 2012. Mmwr Surveill Summ. (2016) 65:1–23. doi: 10.15585/mmwr.ss6503a1, PMID: 27031587 PMC7909709

[2] Maenner MJ Warren Z Williams AR Amoakohene E Bakian AV Bilder DA . Prevalence and characteristics of autism spectrum disorder among children aged 8 years - autism and developmental disabilities monitoring network, 11 Sites, United State. Mmwr Surveill Summ. (2023) 72:1–14. doi: 10.15585/mmwr.ss7202a1, PMID: 36952288 PMC10042614

[3] Diamond A . Executive functions. Annu Rev Psychol. (2013) 64:135–68. doi: 10.1146/annurev-psych-113011-143750, PMID: 23020641 PMC4084861

[4] Demetriou E DeMayo M Guastella A . Executive function in autism spectrum disorder: history, theoretical models, empirical findings, and potential as an endophenotype. Front Psychiatry. (2019) 10:753. doi: 10.3389/fpsyt.2019.00753, PMID: 31780959 PMC6859507

[5] Macoun S Schneider I Bedir B Sheehan J Sung A . Pilot study of an attention and executive function cognitive intervention in children with autism spectrum disorders. J Autism. Dev Disord. (2021) 51:1–11. doi: 10.1007/s10803-020-04723-w, PMID: 33029666

[6] Gusnard DA Akbudak E Shulman GL Raichle ME . Medial prefrontal cortex and self-referential mental activity: Relation to a default mode of brain function. Proc Natl Acad Sci USA. (2001) 98:4259–64. doi: 10.1073/pnas.071043098, PMID: 11259662 PMC31213

[7] Liu Z Cai K Zhu L Xiong X Dong X Pang L . Effects of exercise intervention on executive function and default network functional connectivity in children with autism with mental retardation. J Capital Inst Phys Educ. (2023) 35:493–502. doi: 10.14036/j.cnki.cn11-4513.2023.05.004

[8] O’Hagan AD Behan S Peers C Belton S O’Connor N Issartel J . Do our movement skills impact our cognitive skills? Exploring the relationship between cognitive function and fundamental movement skills in primary school children. J Sci Med Sport. (2022) 25:871–7. doi: 10.1016/j.jsams.2022.08.001, PMID: 36064502

[9] Cristofori I Cohen-Zimerman S Grafman J . Executive functions. Handb. Clin Neurol. (2019) 163:197–219. doi: 10.1016/B978-0-12-804281-6.00011-2, PMID: 31590731

[10] Li L Huang Z Yang Y Wu X . Effects of ball physical activity intervention on basic motor skills and executive function in children with attention deficit hyperactivity disorder. Chin Rehabil. Theory Pract. (2024) 30:479–86. doi: 10.3969/j.issn.1006-9771.2024.04.014

[11] Chen AG Feng L Zhu LN Yan J . Effects of different durations of moderate-intensity basketball exercise on children’s executive function. J Capital Inst Phys Educ. (2015) 27:223–7. doi: 10.14036/j.cnki.cn11-4513.2015.03.007

[12] Chan YS Jang JT Ho CS . Effects of physical exercise on children with attention deficit hyperactivity disorder. Biomed J. (2022) 45:265–70. doi: 10.1016/j.bj.2021.11.011, PMID: 34856393 PMC9250090

[13] Dong X Chen A Liu Z Wang J Cai K Xiong X . Effects of mini-basketball on repetitive stereotyped behaviors and brain gray matter volume in preschool children with autism. Chin Sports Sci Technol. (2020) 56:25–31. doi: 10.16470/j.csst.2020126

[14] Wu Y Ding L Zhang Q Dong Y Tao C Li Z . The effect of physical exercise therapy on autism spectrum disorder: a systematic review and meta-analysis. Psychiatry Res. (2024) 339:116074. doi: 10.1016/j.psychres.2024.116074, PMID: 38986177

[15] Liang X Li R Wong SH Sum RK Wang P Yang B . The effects of exercise interventions on executive functions in children and adolescents with autism spectrum disorder: a systematic review and meta-analysis. Sports Med. (2022) 52:75–88. doi: 10.1007/s40279-021-01545-3, PMID: 34468951

[16] Zhang Y Tian H Tao Y Li Y Wang D Qin L . A study on the effects of three game intervention programs on executive functions of preschool autistic children. Int J Dev Disabil. (2025) 71:168–78. doi: 10.1080/20473869.2023.2215606, PMID: 39882406 PMC11774177

[17] Zhu P Shi Y Yin H . Performance in time-distance replication task and its cognitive mechanisms in depressed patients. Chin J Clin Psychol. (2024) 32:499–504 + 510. doi: 10.16128/j.cnki.1005-3611.2024.03.003

[18] Davidson MC Amso D Anderson LC Diamond A . Development ofcognitive control and executive functions from 4 to 13 years: evidencefrom manipulations of memory, inhibition, and task switching. Neuropsychologia. (2006) 44:2037–78. doi: 10.1016/j.neuropsychologia.2006.02.006, PMID: 16580701 PMC1513793

[19] Zhang JH Lu JC Liu M Yang X . Effects of moderate-intensity gymnastic exercise on executive function in 5 to 6-year-old children. Chin Sch. Health. (2024) 45:326–329 + 334. doi: 10.16835/j.cnki.1000-9817.2024082

[20] Van Riper SM Tempest GD Piccirilli A Ma Q Reiss AL . Aerobic exercise, cognitive performance, and brain activity inadolescents with attention-deficit/hyperactivity disorder. Med Sci Sports Exerc. (2023) 55:1445–55. doi: 10.1249/MSS.0000000000003159, PMID: 36897828

[21] Schopler E Reichler RJ DeVellis RF Daly K . Toward objective classification of childhood autism: Childhood Autism Rating Scale (CARS). J Autism Dev Disord. (1980) 10:91–103. doi: 10.1007/BF02408436, PMID: 6927682

[22] Brst JR . Effects of physical activity on children’s executive function: contributions of experimental research on aerobic exercise. Dev Rev. (2010) 30:331–551. doi: 10.1016/j.dr.2010.07.001, PMID: 21818169 PMC3147174

[23] Song Y Ren Y Zhu F Kuang D Cao Q Lin Y . Characteristics and relationship between gross motor skills and executive function development in children with attention deficit hyperactivity disorder. Chin Rehabil. Theory Pract. (2024) 30:1–9. doi: 10.3969/j.issn.1006-9771.2024.01.001

[24] Leisman G Moustaf AA Shafir T . Thinking, walking, talking: integratory motor and cognitive brain function. Front Public Health. (2016) 4:94. doi: 10.3389/fpubh.2016.00094, PMID: 27252937 PMC4879139

[25] Chen X Liang C Li M Wang Q Sun M . Effects of aerobic exercise on core symptoms and executive function in children with attention deficit hyperactivity disorder. Chin Rehabil. Theory Pract. (2022) 28:704–9. doi: 10.3969/j.issn.1006-9771.2022.06.012

[26] Chaddock-Heyman L Erickson KI Holtrop JL Voss MW Pontifex MB Raine LB . Aerobic fitness is associated with greater white matter integrity in children. Front Hum Neurosci. (2014) 8:584. doi: 10.3389/fnhum.2014.00584, PMID: 25191243 PMC4137385

[27] Cai CX Zhang YL . Research progress on the mechanism of exercise to improve brain executive function. J Chengdu Inst Phys Educ. (2019) 45:120–6. doi: 10.15942/j.jcsu.2019.06.020

[28] Chen JW Zhu K . Single exercise for core symptoms and executive functions in ADHD: a systematic review and meta-analysis. J Attent. Disord. (2024) 28:399–414. doi: 10.1177/10870547231217321, PMID: 38156611

[29] Mangerud WL Bjerkeset O Lydersen S Indredavik MS . Physical activity in adolescents with psychiatric disorders and in the general population. Child Adolesc. Psychiatry Ment Health. (2014) 8:2. doi: 10.1186/1753-2000-8-2, PMID: 24450542 PMC3914726

[30] Abdulghani A Poghosyan M Mehren A Philipsen A Anderzhanova E . Neuroplasticity to autophagy cross-talk in a therapeutic effectof physical exercises and irisin in ADHD. Front Mol Neurosci. (2023) 26:997054. doi: 10.3389/fnmol.2022.997054, PMID: 36776770 PMC9909442

[31] Adolph KE Hoch JE . Motor development: embodied, embedded, enculturated, and enabling. Annu Rev Psychol. (2019) 70:141–64. doi: 10.1146/annurev-psych-010418-102836, PMID: 30256718 PMC6320716

[32] Bayazit B . The effects of basketball basic skills training on gross motor skills development of female children. Educ Res Rev. (2015) 10:648–53. doi: 10.5897/err2014.2020

[33] Berwid OG Halperin JM . Emerging support for a role of exercise in attention-deficit/hyperactivity disorder intervention planning. Curr Psychiatry Rep. (2012) 14:543–51. doi: 10.1007/s11920-012-0286-0, PMID: 22895892 PMC3724411

[34] Chen A . Physical education innovation in the vision of educational neuroscience. Beijing: Educational Science Press (2016).

[35] Diamond A Lee K . Interventions shown to aid executive function development in children 4 to 12 years old. Science. (2011) 333:959–64. doi: 10.1126/science.1204529, PMID: 21852486 PMC3159917

[36] Wang JG Cai KL Liu ZM Herold F Zou L Zhu LN . Effects of mini-basketball training program on executive functions and core symptoms among preschool children with autism spectrum disorders. Brain Sci. (2020) 10:263. doi: 10.3390/brainsci10050263, PMID: 32365853 PMC7287705

[37] Hu M Zhou H Bao X Wang J . Effects of aerobic exercise on fatigue during chemotherapy in elderly patients with Malignant tumors based on the theory of movement and yang generation. Evid. Based Nurs. (2024) 10:3790–4. doi: 10.12102/j.issn.2095-8668.2024.20.035

[38] Ma J Bu SM Cheng Y . Role and mechanism of exercise-generated lactate in the nervous system. Adv Biochem Biophys. (2025) 52:348–57. doi: 10.16476/j.pibb.2024.0010

